# Inhibition of stearoyl-CoA desaturase 1 in the mouse impairs pancreatic islet morphogenesis and promotes loss of β-cell identity and α-cell expansion in the mature pancreas

**DOI:** 10.1016/j.molmet.2022.101659

**Published:** 2022-12-15

**Authors:** Aneta M. Dobosz, Justyna Janikiewicz, Ewelina Krogulec, Anna Dziewulska, Anna Ajduk, Marcin Szpila, Hanna Nieznańska, Andrzej A. Szczepankiewicz, Dorota Wypych, Agnieszka Dobrzyn

**Affiliations:** 1 Laboratory of Cell Signaling and Metabolic Disorders, Nencki Institute of Experimental Biology, Polish Academy of Sciences, Warsaw, Poland; 2 Department of Embryology, Institute of Developmental Biology and Biomedical Sciences, Faculty of Biology, University of Warsaw, Warsaw, Poland; 3 Laboratory of Electron Microscopy, Nencki Institute of Experimental Biology, Polish Academy of Sciences, Warsaw, Poland

**Keywords:** SCD1, Pancreatic islets, Alpha cells, Beta cells, DNA methylation, Insulin, Glucagon, β-Cell identity, Pancreas embryogenesis, Lipotoxicity, Type 2 diabetes

## Abstract

Abnormalities that characterize the pathophysiology of type 2 diabetes (T2D) include deficiencies of β-cells and the expansion of α-cells in pancreatic islets, manifested by lower insulin release and glucagon oversecretion. The molecular mechanisms that determine intra-islet interactions between pancreatic α- and β-cells are still not fully understood. The present study showed that stearoyl-coenzyme A (CoA) desaturase 1 (SCD1), an enzyme that is implicated in fatty acid metabolism, serves as a checkpoint in the control of endocrine cell equilibrium in pancreatic islets. Our data showed that SCD1 activity is essential for proper α-cell and β-cell lineage determination during morphogenesis of the pancreas and the maintenance of mature β-cell identity. The inhibition of SCD1 expression/activity led to both a decrease in the expression of β-cell signature genes (e.g., *Pdx1*, *Nkx6.1*, *MafA*, and *Neurod1*, among others) and induction of the expression of the dedifferentiation marker *Sox9* in mature pancreatic islets. The transcriptional repression of *Pdx1* and *MafA* in SCD1-deficient β-cells was related to the excessive methylation of promoter regions of these transcription factors. In contrast, SCD1 ablation favored the formation of α-cells over β-cells throughout pancreas organogenesis and did not compromise α-cell identity in adult pancreatic islets. Such molecular changes that were caused by SCD1 downregulation resulted in the mislocalization of α-cells within the core of islets and increased the ratio of pancreatic α- to β-cell mass. This was followed by islet dysfunction, including impairments in glucose-stimulated insulin release, simultaneously with elevations of basal glucagon secretion. Altogether, these findings provide additional mechanistic insights into the role of SCD1 in the pathogenesis of T2D.

## Highlights

• SCD1 deficiency promotes α-cell over β-cell formation during pancreas morphogenesis.
• SCD1 determines maintenance of α/β-cell equilibrium in mature pancreatic islets.
• SCD1 protects β-cells from lipid-derived loss of identity.
• Expression of key β-cell transcription factors is regulated by SCD1 via epigenetic mechanisms.

## Graphical abstract

**Figure:**
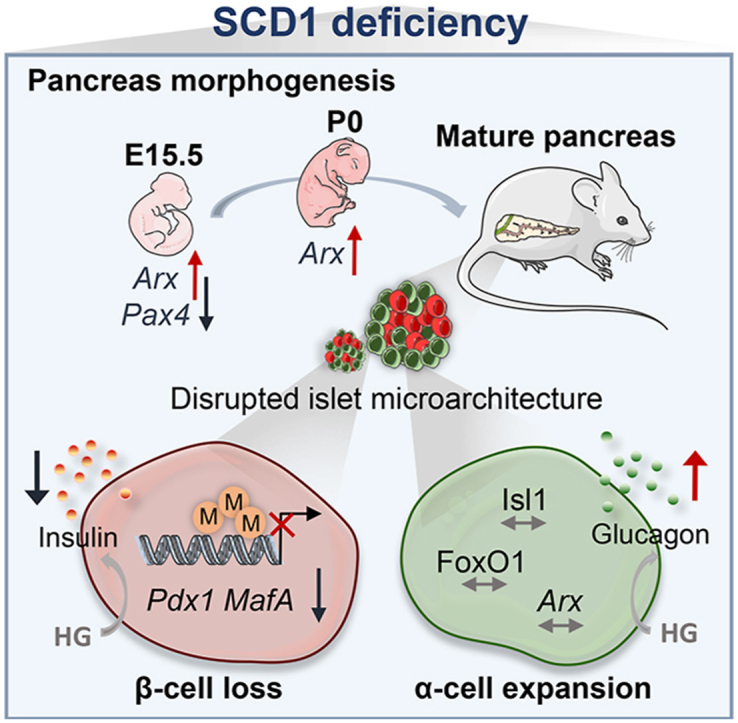

## 1 Introduction

Type 2 diabetes (T2D) is a chronic metabolic condition that is characterized by impairments in blood glucose homeostasis that result from insulin resistance and pancreatic islet dysfunction [1]. T2D has been considered a “bihormonal disorder.” The improper function of glucagon-secreting α-cells and insulin-secreting β-cells in pancreatic islets is central for onset and progression of the disease [[2], [3], [4]]. Multiple mechanisms that lead to reductions of the number and insulin-secretory capacity of β-cells, with the simultaneous expansion of α-cells followed by excessive glucagon release in T2D patients, have been proposed. Theories range from low postnatal β-cell mass that is caused by genetic or environmental factors, to the compromised capability for β-cell compensation and massive β-cell death [3,5].

Interestingly, recent studies highlight a degree of α- and β-cell plasticity under conditions of metabolic stress, suggesting that the loss of mature β-cell identity is critical for pancreatic islet failure in T2D [[5], [6], [7], [8]]. The molecular identity and unique functionality of pancreatic α- and β-cells are defined by the expression of specific transcription factors (TFs) that govern pancreatic islet morphogenesis and the specification of endocrine cell lineages during embryonic development. In adulthood, in turn, these TFs actively regulate insulin and glucagon gene expression, secretory granule formation, and exocytosis [[9], [10], [11]]. Disruption of the network of these TFs leads to (*i*) the dedifferentiation of α- and β-cells (i.e., conversion of mature α- or β-cells to the progenitor-like state), (*ii*) β-to-α cell and α-to-β cell transdifferentiation, or (*iii*) the formation of bihormonal, intermediate cells that exhibit features of both α- and β-cells (e.g., the expression of both insulin and glucagon in parallel) [12].

A direct contribution of specific TFs in both the process of α- and β-cell embryonic differentiation and the determination of mature α- and β-cell identity has been investigated in murine models [5,10]. Three transitions define pancreatic organogenesis in rodents, whereas pancreatic α- and β-cell lineage specification and allocation occur during secondary (embryonic day 12.5 [E12.5] to E16.5) and tertiary (E16.5-postnatal) transition [13]. The master regulator of pancreatic endocrine progenitor cell formation is neurogenin 3 (NGN3). As differentiation proceeds, specialization toward β-cell fate depends on the expression of such TFs as pancreatic and duodenal homeobox 1 (PDX1), NK6 homeobox 1 (NKX6-1), v-maf musculoaponeurotic fibrosarcoma oncogene homolog A (MAFA), neuronal differentiation 1 (NEUROD1), and paired box 4 (PAX4) [10]. In contrast, higher expression levels of aristaless-related homeobox (ARX) favors the formation of α-cells [14]. The group of TFs whose stable expression determines both α- and β-cell differentiation and the maintenance of mature identity also includes LIM homeobox protein islet 1 (ISL1) [15], forkhead box protein O1 (FOXO1) [16], NK2 homeobox 2 (NKX2.2) [17], and paired box 6 (PAX6) [18]. Importantly for the concept of the metabolic regulation of α- and β-cell identity, many of the TFs that are mentioned above (e.g., PDX1, NKX6.1, MAFA, PAX6, and FOXO1) are inactivated by hyperglycemia and lipotoxicity, pathological conditions that are associated with T2D [5].

Stearoyl-coenzyme A (CoA) desaturase 1 (SCD1) is a lipogenic enzyme that catalyzes the synthesis of monounsaturated fatty acids (MUFAs), mainly palmitoleate (16:1) and oleate (18:1n9), from saturated fatty acids (SFAs), palmitate (16:0) and stearate (18:0), respectively [19]. Because of beneficial effects of SCD1 deficiency that have been demonstrated in mouse models (e.g., lower adiposity and improvements in glucose tolerance), SCD1 represents a potential target to resolve obesity-related metabolic diseases [19]. Paradoxically, however, the unqualified inhibition of SCD1 acts as a double-edged sword in pancreatic islets through mechanisms that remain largely unknown. The lower activity of SCD1 and the resulting oversupply of toxic SFAs contribute to β-cell failure and the development of T2D [[19], [20], [21]]. The inhibition of SCD1 activity leads to various deleterious effects in β-cells, such as the induction of endoplasmic reticulum stress, mitochondrial damage, the reduction of insulin secretion, abnormal autophagy, and the disruption of integrity of the cellular membrane [22,23]. Additionally, our recent data showed that SCD1 is also involved in the epigenetic regulation of gene expression in pancreatic β-cells, adipocytes, and skeletal muscles [[24], [25], [26]].

To dissect the molecular mechanisms that underpin pancreatic islet failure upon SCD1 deficiency, the present study investigated the involvement of SCD1 in α- and β-cell differentiation during pancreas morphogenesis and the role of SCD1 in maintaining α- and β-cell identity in mature pancreatic islets. We demonstrated that SCD1 ablation promotes the formation and survival of α-cells over β-cells in pancreatic islets. The mechanistic implications of SCD1 deficiency in pancreatic islets included a methylation-dependent decrease in expression of β-cell-specific TFs that resulted in the loss of β-cell identity. Instead, α-cell identity remained unaltered upon SCD1 knockout. It consequently gave rise to a shift in the balance from β-cells toward α-cells in pancreatic islets, the disruption of islet microarchitecture, and islet dysfunction.

## 2 Results

### 2.1 SCD1 deficiency promotes α-cell over β-cell formation during mouse pancreatic islet morphogenesis

**Figure 1:**
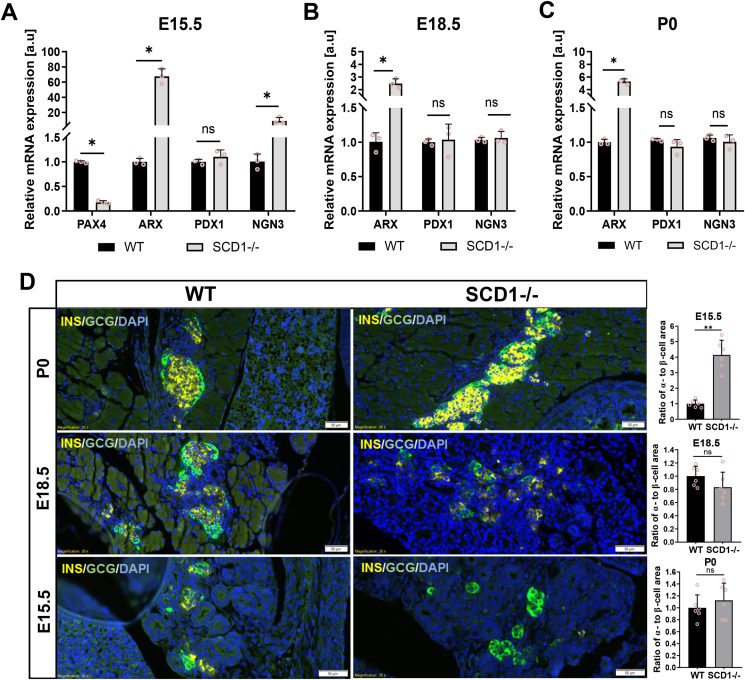
**Depletion of *Scd1* enhances α-cell formation in fetal mouse pancreas.** Results of the quantification by qRT-PCR of *Pdx1, Arx, Pax4*, and *Ngn3* mRNAs in WT and SCD1−/− pancreas on E15.5 **(A)**, E18.5 **(B)**, and P0 (neonatal mice) **(C)**. Insulin (yellow) and glucagon (green) immunofluorescent staining of paraffin-embedded pancreas sections from E15.5, E18.5, and P0 WT and SCD1−/− mice with summary graphs of calculated ratio of α-cell area to β-cell area **(D)**. Scale bars = 50 μm. Cell nuclei were stained with 4′,6-diamidino-2-phenylindole (DAPI). The data are representative of *n* = 3 mice/group. The data are expressed as mean ± SD. *∗p* < 0.05, *vs*. WT.

In our primary experiments, to gain insights into the role of SCD1 in α-cell and β-cell neogenesis, we investigated the expression of crucial genes that are involved in α- and β-cell reprogramming in the developing pancreas in wildtype (WT) and SCD1−/− mice. The analyses were performed at critical time points of endocrine cell lineage differentiation: E15.5 (when the major burst in β-cell generation occurs and when the highest expression of the endocrine progenitor marker NGN3 is detected), E18.5 (when cells undergo further remodeling, maturation, and clustering into islets), and after birth (postnatal day 0 [P0], during the ultimate process of mature organ establishment) [13,[27], [28], [29]]. Interestingly, the deletion of *Scd1* caused a ∼200-times increase in the expression of the α-cell signature gene *Arx* and dramatically reduced by > 80% expression of the β-cell key factor *Pax4* in the E15.5 pancreas compared with WT (Figure 1A). The expression of *Pax4* was undetected in the pancreas in WT and SCD1−/− embryos at later stages of pancreas organogenesis, whereas the expression of *Arx* remained elevated in pancreatic tissue from E18.5 and P0 SCD1−/− animals. *Arx* mRNA levels were ∼2.5-fold higher on E18.5 and ∼five-fold higher on P0 in the pancreas in SCD1−/− buds compared with WT (Figure 1B, C). In contrast, we found that the level of mRNA of the β-cell marker *Pdx1* in pancreatic tissue in E15.5, E18.5, and P0 SCD1−/− animals was not significantly different from their WT counterparts (Figure 1A–C). In turn, *Ngn3* gene expression increased ∼8-times in the pancreas in E15.5 SCD1−/− mice compared with WT mice. Instead, the abundance of *Ngn3* mRNA on E18.8 and P0 in the pancreas in SCD1−/− mice was sustained at a similar level as in WT mice (Figure 1A–C).

We also analyzed α- and β-cell lineage formation on pancreatic sections from E15.5, E18.5, and P0 WT and SCD1−/− animals using double-immunofluorescence staining for glucagon and insulin. We found a significant β-cell to α-cell shift on E15.5 upon the loss of SCD1. The ratio of the α-cell area to β-cell area increased four times in pancreatic tissue from E15.5 SCD1−/− mice compared with WT mice. However, we did not find significant differences between α-to-β-cell ratios in the E18.5 and P0 SCD1-deficient pancreas compared with WT (Figure 1D).

### 2.2 SCD1 knockdown reduces the expression of β-cell-specific TFs, upregulates the expression of α-cell-specific TFs, and triggers expression of the dedifferentiation marker Sox9 in mature mouse pancreatic islets

**Figure 2:**
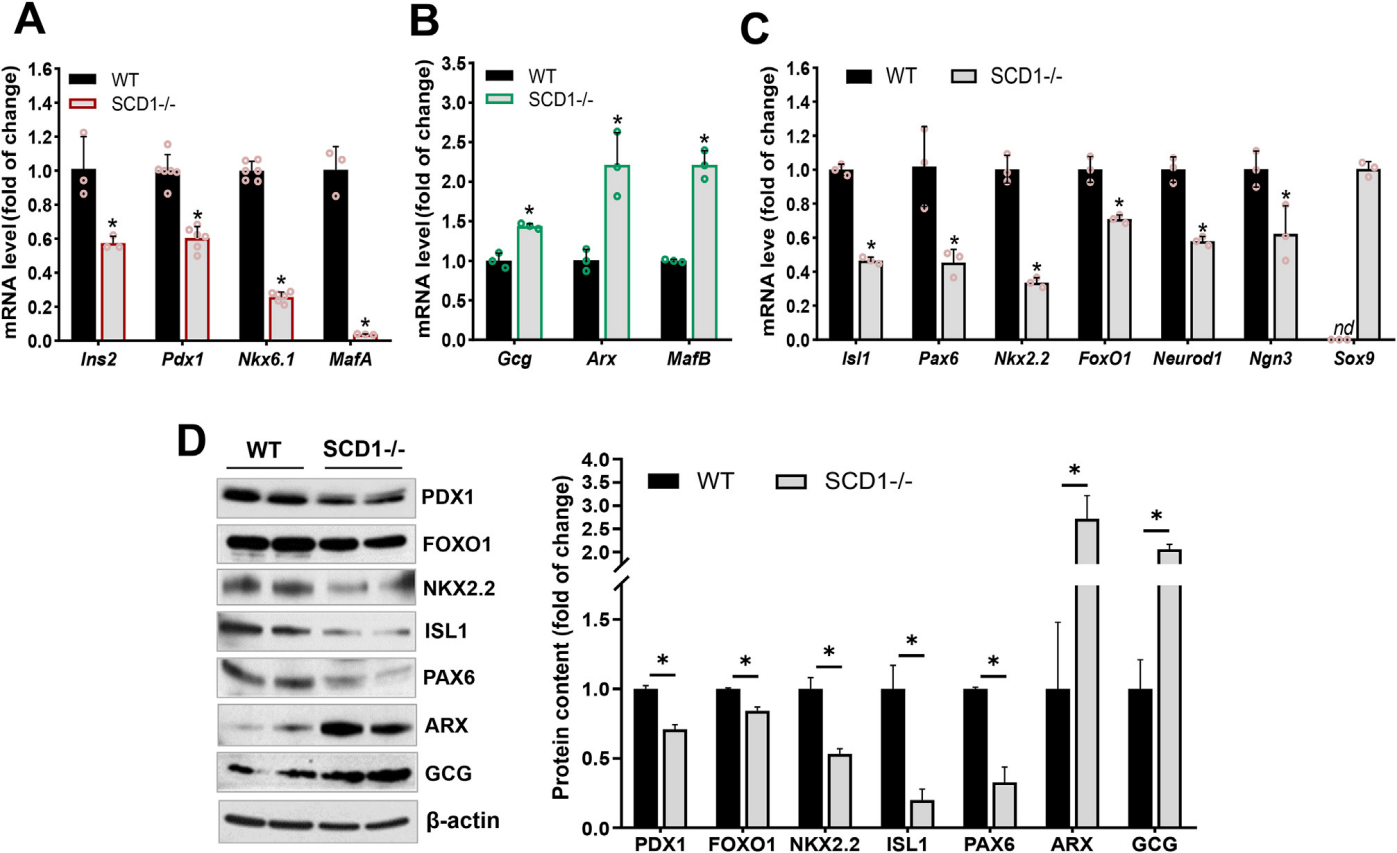
**Pancreatic islets in SCD1−/− mice exhibit alterations of the expression of TFs that are involved in maintaining α-cell and β-cell identity.** mRNA levels of **(A)** β-cell-specific genes (*Ins2*, *Pdx1*, *Nkx6.1*, *MafA*), **(B)** α-cell-specific genes (*Gcg*, *Arx*, *MafB*), **(C)** both α-cell and β-cell signature genes (*Isl1*, *Pax6*, *Nkx2.2*, *FoxO1*, *Neurod1*, *Ngn3*), and dedifferentiation marker *Sox9* in pancreatic islets from 10-week-old control (WT) and SCD1−/− mice. **(D)** Immunoblot analysis of GCG, PDX1, FOXO1, NKX2.2, ISL1, PAX6, and ARX proteins in pancreatic islet lysates from WT and SCD1−/− mice: representative immunoblots and quantitative, densitometric analysis of Western blot bands. β-actin was used as a loading control. The data are representative of *n* = 6 mice/group. The data are expressed as mean ± SD. *∗p* < 0.05, *vs*. WT. nd, not detected.

To identify primary molecular changes that can affect α- and β-cell identity in adult mouse pancreatic islets, we validated the transcription level of α- and β-cell essential genes in isolated islets from 10-week-old SCD1-deficient mice. The knockdown of *Scd1* led to a decrease in the expression of genes that encode TFs that control β-cell identity and directly regulate the insulin gene promoter [11], such as *Pdx1*, *Nkx6.1*, and *MafA* (mRNA expression downregulated by ∼40%, ∼75%, ∼95%, respectively) compared with pancreatic islets from WT mice (Figure 2A). Conversely, mRNA levels of the α-cell-specific markers *Arx* and *MafB* were more than two-fold higher in pancreatic islets from SCD1−/− mice compared with WT (Figure 2B). In SCD1−/− pancreatic islets, lower levels of mRNA of the *Isl1* (∼55%), *Pax6* (∼65%), *Nkx2.2* (∼55%), and *Neurod1* (∼40%) genes were also detected (Figure 2C). Moreover, in SCD1−/− pancreatic islets, there was a ∼30% decrease in the expression of *Ngn3* and ∼20% decrease in the expression of *FoxO1* compared with WT (Figure 2C). SCD1-deficient islets were also characterized by a ∼45% reduction of transcription of the *Ins2* gene, which encodes insulin, and a simultaneous ∼45% increase in the level of the *Gcg* transcript, which encodes glucagon, compared with WT islets (Figure 2A, B).

We also validated a subset of these gene changes in mouse islets at the protein level. Consistent with mRNA levels, a two-fold increase in glucagon (GCG) content and ∼2.5-fold increase in protein levels of ARX were observed in pancreatic islets from SCD1−/− mice compared with WT (Figure 2D). In contrast, in pancreatic islets, SCD1 deletion reduced expression of the β-cell-specific factor PDX1 by ∼25% and significantly decreased expression of the TFs NKX2.2, ISL1, PAX6, and FOXO1 by ∼50%, ∼80%, ∼60%, and ∼15%, respectively, compared with WT (Figure 2D).

To verify whether SCD1 knockout disrupts islet cell identity and triggers the regression of mature α- and β-cells from a specialized function, we determined the expression of dedifferentiation markers in pancreatic islets from WT and SCD1−/− mice. Interestingly, among the other tested genes, *Sox9* mRNA was detected in pancreatic islets from SCD1−/− mice (Figure 2C). The expression of *Sox9* is a characteristic of pre-endocrine progenitor cells, pancreatic duct cells, and dedifferentiating β-cells [[30], [31], [32]]. The *Sox9* gene was not expressed in pancreatic islets from WT mice (Figure 2C).

### 2.3 SCD1 affects the level of identity markers in pancreatic INS-1E cells but not in αTC1-6 cells

**Figure 3:**
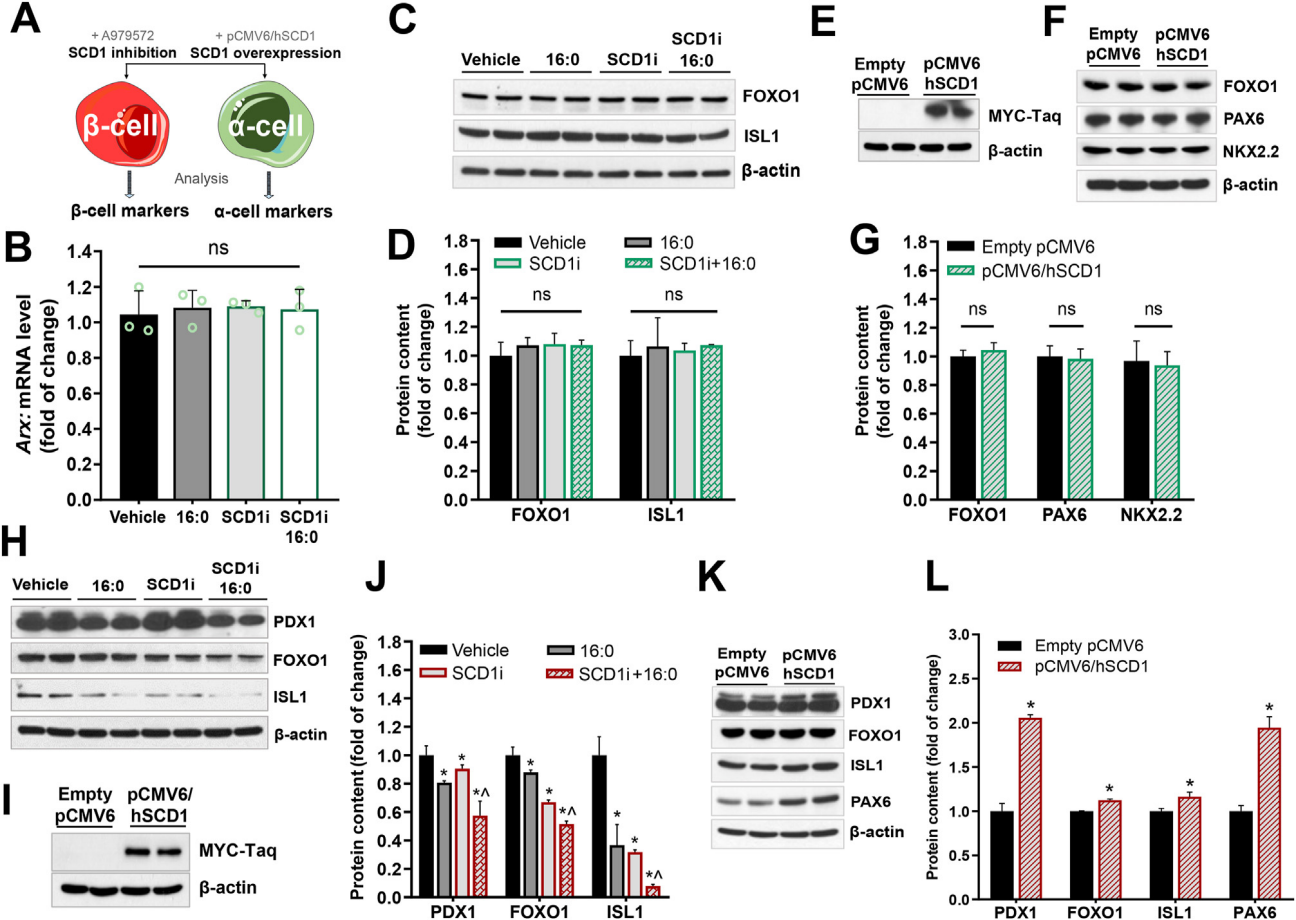
**SCD1 expression/activity determines the expression level of signature TFs in pancreatic INS-1E cells but does not affect the expression of signature TFs in pancreatic αTC1-6 cells. (A)** Schematic design of the *in vitro* study of the effects of SCD1 inhibition/overexpression on the levels of α-cell- and β-cell-specific TFs. **(B**–**D)** mRNA levels of *Arx***(B)** and Western blot analysis of the abundance of FOXO1 and PAX6 proteins **(C, D)** in αTC1-6 cells that were incubated with the SCD1 inhibitor (SCDi) and/or subsequently co-treated with 16:0. **(E**–**G)** Representative immunoblots that show levels of recombinant SCD1 protein fused with Myc-Taq **(E)** and content of FOXO1, PAX6, and NKX2.2 proteins **(F, G)** in αTC1-6 cells that transiently overexpressed SCD1. **(H, J)** Evaluation of PDX1, FOXO1, and ISL1 protein content in INS-1E cells that were treated with the SCD1 inhibitor and/or palmitic acid (16:0): **(H)** representative immunoblots, **(J)** quantitative, densitometric analysis of Western blot bands. *∗p* < 0.05, *vs*. vehicle; ^*ˆ*^*p* < 0.05, vs. SCD1i. **(I, K, L)** Level of recombinant SCD1 protein fused with Myc-Taq **(I)**, PDX1, FOXO1, NKX2.2, ISL1, and PAX6 proteins **(K)**, and densitometric validation of the abundance of these proteins **(L)** in INS-1E cells that transiently overexpressed SCD1 (transfected with pCMV6/hSCD1 plasmid) and control INS-1E cells (transfected with empty vector pCMV6). *∗p* < 0.05, *vs*. empty pCMV6. β-actin was used as a loading control. The data are expressed as mean ± SD. *n* = 3. ns, nonsignificant.

In the next step of the experiments, we investigated the influence of SCD1 activity/expression on the level of signature TFs directly in α-cells and β-cells. We examined the protein abundance of selected TFs that are involved in maintaining the mature α- and β-cell state after SCD1 inhibition and SCD1 overexpression in rat INS-1E and mouse αTC1-6 cell lines (Figure 3A). To induce lipotoxicity and accentuate the effects of SCD1 deficiency [33,34], the cells were treated with 16:0.

The analyses showed that SCD1 inhibition decreased the protein content of PDX1 by ∼20%, FOXO1 by ∼12%, and ISL1 by ∼60% in INS-1E cells compared with control (Figure 3H, J). In the groups of cells that were incubated simultaneously with the SCD1 inhibitor and 16:0, the abundance of PDX1, FOXO1, and ISL1 was ∼30%, 50%, and 85% lower, respectively, than in the control group of cells (Figure 3H, J). The temporal overexpression of recombinant SCD1 protein in INS-1E cells that were transfected with the pCMV6 plasmid that carries the sequence of the human *SCD1* gene (hSCD1) was confirmed by Myc-Taq detection (Figure 3I). The levels of such TFs as PDX1 and PAX6 increased in INS-1E cells that overexpressed hSCD1 by ∼2-fold, and the content of FOXO1, NKX.2.2, and ISL1 increased by ∼10%, ∼25%%, and ∼15%, respectively, compared with the control group of cells that were transfected with an empty vector (Figure 3K, L).

In contrast to INS-1E cells, neither SCD1 inhibition nor SCD1 overexpression in αTC1-6 cells affected the level of TFs that are responsible for maintaining specific features of α-cells. The expression level of the α-cell-specific *Arx* gene was unchanged in αTC1-6 cells that were treated with the SCD1 inhibitor and 16:0 compared with the control group (Figure 3B). Furthermore, in SCD1-deficient αTC1-6 cells that were stimulated with 16:0, the content of such TFs as FOXO1 and ISL1 remained similar to control cells (Figure 3C, D). Likewise, SCD1 inhibition and hSCD1 overexpression in αTC1-6 cells did not induce significant changes in levels of FOXO1, PAX6, or NKX2.2 compared with control cells that were transfected with the empty pCMV6 plasmid (Figure 3E–G).

### 2.4 Lower expression of β-cell signature genes Pdx1 and MafA in SCD1-deficient INS-1E cells corresponds with the higher methylation of promoter regions of these genes

**Figure 4:**
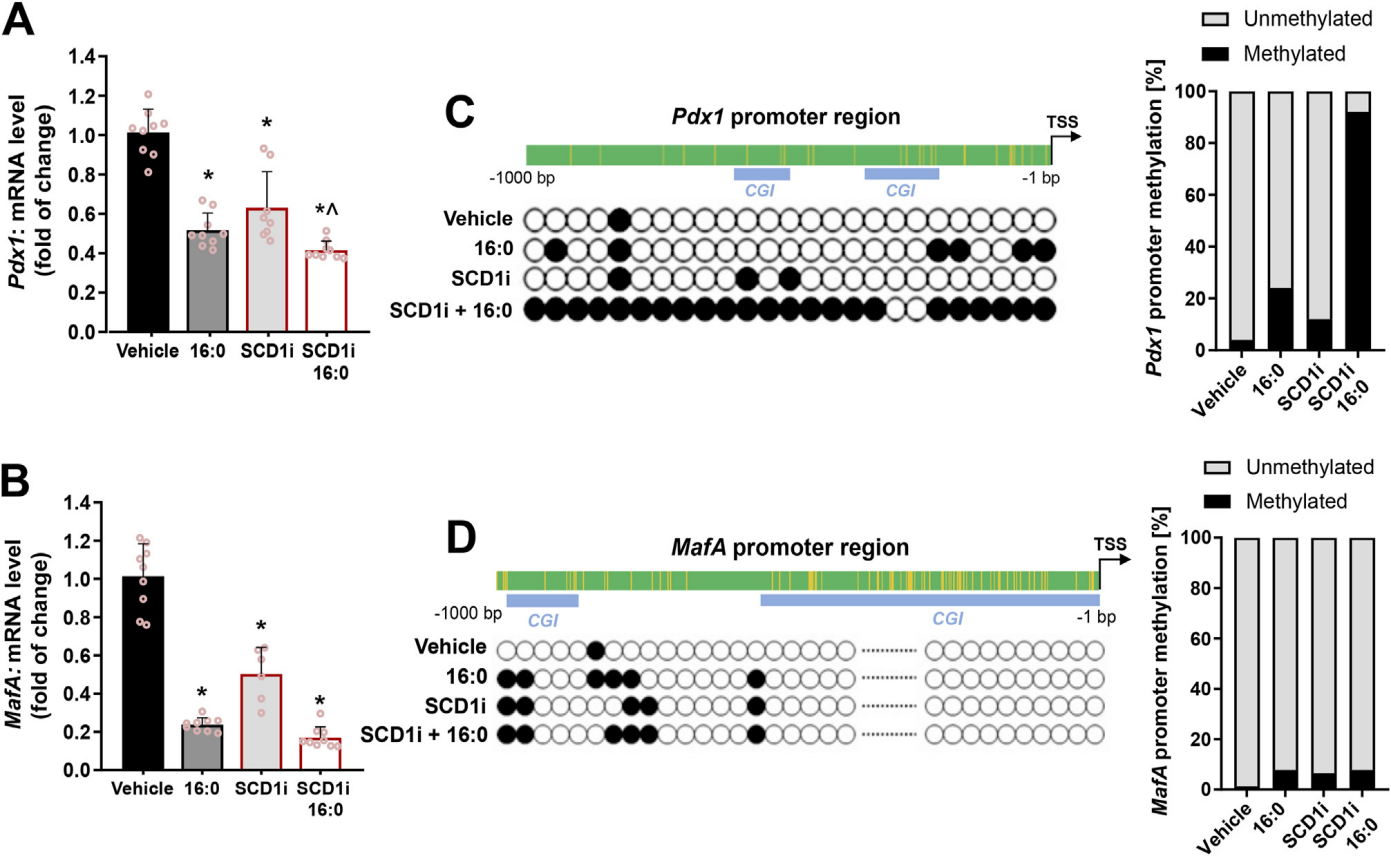
**SCD1 regulates the methylation status of CpG sites in *Pdx1* and *MafA* gene promoters in pancreatic INS-1E cells. (A, B)** mRNA levels of *Pdx1***(A)** and *MafA***(B)** genes in INS-1E cells after SCD1 inhibition (SCD1i) and/or subsequently co-treated with palmitate (16:0). *∗p* < 0.05, *vs*. vehicle; *ˆp* < 0.05, *vs*. SCD1i. The data are expressed as the mean ± SD from three independent experiments. **(C–F)** Bisulfite sequencing analysis of *Pdx1* and *MafA* promoter regions (−1000 bp to 0 bp from transcription start site): methylation status and distribution of 5-methylcytosines alongside *Pdx1***(C)** and *MafA***(D)** promoter sequences, rate of cytosine DNA methylation in *Pdx1* and *MafA* promoters in INS-1E cells after SCD1 inhibition (SCD1i) and/or palmitate (16:0) treatment. Blue boxes indicate CpG islands (regions enriched in CG dinucleotides) in the DNA sequence. *n* = 3. TSS, transcription start site.

A decrease in the abundance of β-cell-specific TFs was observed in rat INS-1E cells upon SCD1 inhibition and 16:0 treatment at the mRNA level. Expression of the *Pdx1* gene was ∼35% lower and expression of the *MafA* gene was ∼50% lower in SCD1-deficient INS-1E cells than in control cells (Figure 4A, B). *Pdx1* and *MafA* expression was downregulated by more than 40% and 60%, respectively, in INS-1E cells that were treated with 16:0 compared with control (Figure 4A, B). In INS-1E cells that were incubated with the SCD1 inhibitor in combination with 16:0, *Pdx1* mRNA levels decreased by ∼60%, and *MafA* mRNA levels decreased by ∼80–85% compared with control (Figure 4A, B).

An increase in the methylation of cytosine residues within the promoter region correlates with lower levels of gene transcription [35]. To clarify whether lower expression of the *Pdx1* and *MafA* genes is related to the hypermethylation of promoter regions of these genes, bisulfite sequencing analysis was performed. Within the promoter sequence of the *Pdx1* gene (1000 bp upstream of the transcription start site), 25 CpG sites were identified. The calculated percentages of methylation of the analyzed sequence of the *Pdx1* promoter in INS-1E cells were 4% for the control group of cells, 24% for the group of cells that were incubated with 16:0, 12% for the group of cells after SCD1 inhibition, and 92% for the group of cells that were incubated with both 16:0 and SCD1 inhibitor (Figure 4C).

Moreover, 77 potential methylation sites were detected within the *MafA* promoter sequence. Among these, ∼1.3% were methylated under control conditions. The level of methylation of the *MafA* promoter in INS-1E cells slightly increased after SDC1 inhibition and upon 16:0 stimulation. The degree of *MafA* promoter methylation was ∼6.5% for cells that were incubated with the SCD1 inhibitor and ∼7.8% for cells that were incubated with 16:0 or both 16:0 and the SCD1 inhibitor (Figure 4D).

### 2.5 SCD1 knockout results in the mislocation of α-cells and increase in the ratio of α-to-β cells in mouse pancreatic islets

In further experiments, we assessed the implications of SCD1 deficiency on pancreatic islet cytoarchitecture, pancreatic α- and β-cell mass, and the function of these cells in pancreatic islets in adulthood under conditions of metabolic stress that is associated with T2D. To induce lipotoxicity, WT and SCD1−/− mice were fed a high-fat (HF) diet. High-fat diet-fed WT mice developed weight gain and exhibited lower glucose tolerance compared with their normal chow-fed littermates in response to glucose challenge (Figure S1). SCD1−/− animals that were fed the HF diet, because of their favorable metabolic profile [19,20], maintained normal glucose tolerance and were resistant to HF diet-induced obesity (Figure S1).

**Figure 5:**
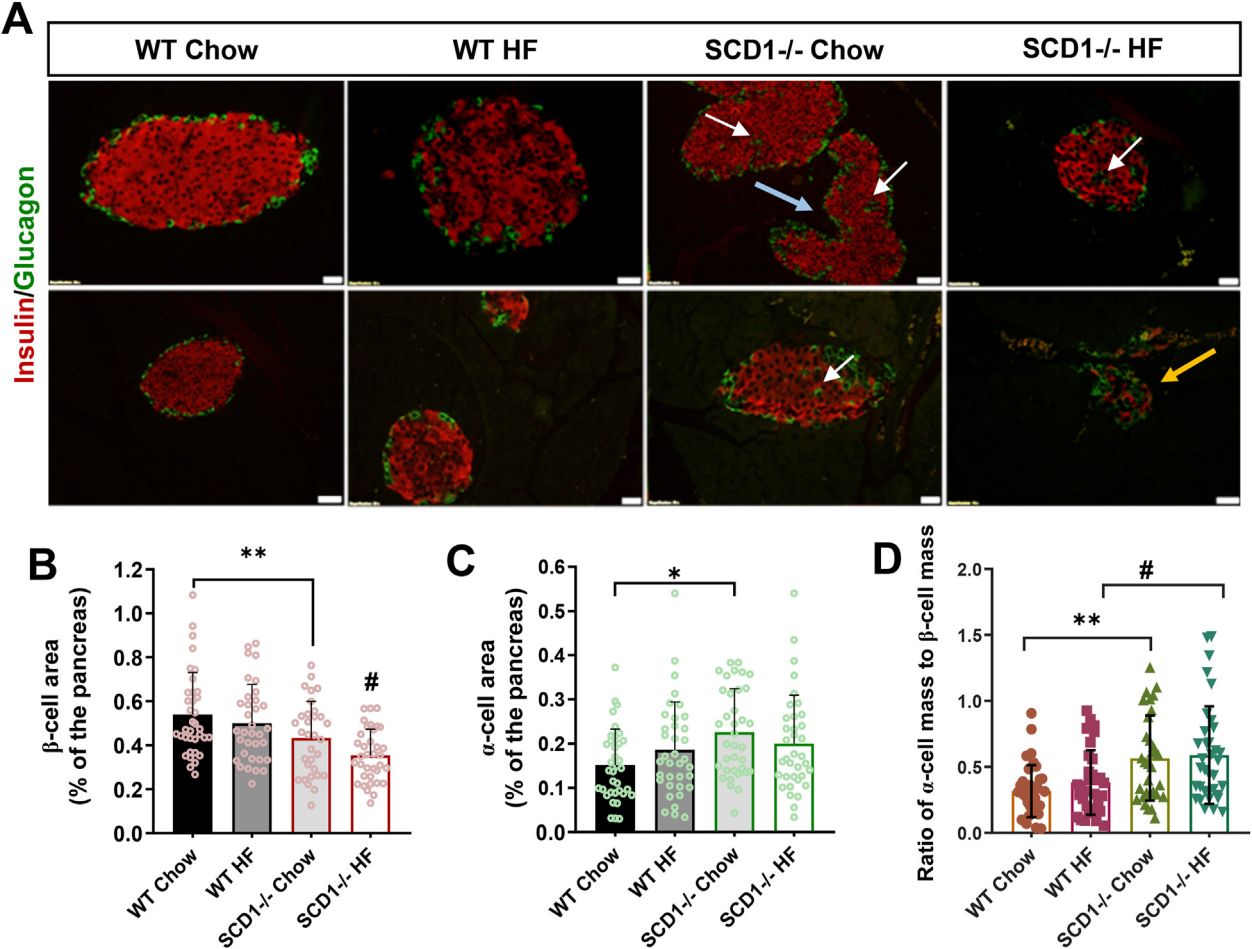
**Effect of SCD1 knockout in mouse on pancreatic islet cytoarchitecture. (A)** Immunofluorescent labeling of insulin-positive cells (red) and glucagon-positive cells (green) in pancreatic islets in WT and SCD1−/− mice that were fed a chow or HF diet, showing the distribution of these cells within islets. The arrows indicate the following: white (α-cells located in the core of the islet), blue (irregular-shaped islet), yellow (disproportionate number of α-cells relative to β-cells within the islet). Images are representative of at least three mice. Scale bars = 20 μm. **(B**–**D)** Morphometric analyses of pancreatic islets in sections of the pancreas from WT and SCD1−/− mice that were fed a chow or HF diet: β-cell area **(B)**, α-cell area **(C)**, and α- to β-cell ratio **(D)**. The data are expressed as mean ± SD. *n* = 3 mice/group. *∗p* < 0.05, *∗∗p* < 0.005, *vs*. WT chow; ^#^*p* < 0.05, *vs*. WT HF.

Regardless of diet, pancreatic islets in WT animals presented a classic cytoarchitecture. Wildtype islets were usually oval, and their core was formed by insulin-secreting β-cells, whereas glucagon-secreting α-cells were localized on the islet periphery (Figure 5A). In contrast, pancreatic islets in SCD1−/− mice were often irregular, and α-cells were also found in the core of islets (Figure 5A). Disturbances in pancreatic islet cytoarchitecture were particularly evident in the group of SCD1−/− mice after they received the HF diet. Moreover, in SCD1−/− islets, the area that was occupied by β-cells was ∼20% smaller, whereas the area that was occupied by α-cells was ∼40% larger compared with WT animals (Figure 5B, C). Similarly, in SCD1−/− HF diet-fed animals, the β-cell area was ∼29% smaller than in WT HF diet-fed animals (Figure 5C). The ratio of α-cell mass to β-cells mass was ∼40% higher in pancreatic islets in SCD1−/− chow-fed animals compared with WT chow-fed animals and ∼35% higher in pancreatic islets in SCD1−/− HF diet-fed animals compared with WT HF diet-fed animals (Figure 5D). In both WT and SCD1−/− mice, administration of the HF diet did not notably affect the ratio of α- to β-cell mass compared with WT and SCD1−/− animals that were fed the chow diet, respectively (Figure 5D).

### 2.6 Knockout of SCD1 in mouse led to the disruption of insulin secretory machinery in β-cells and elevated the synthesis and basal secretion of glucagon from α-cells

To assess α-cell and β-cell populations at the single-cell level in WT and SCD1−/− primary pancreatic islets, transmission electron microscopy (TEM) was performed. Based on the TEM images, the mean size (longer diagonal), maturity, number, and heterogeneity of glucagon- and insulin-containing secretory granules in α-cells and β-cells were evaluated. The analyses were followed by the functional testing of WT and SCD1−/− islets in the presence of high glucose (HG) and low glucose (LG) concentrations.

**Figure 6:**
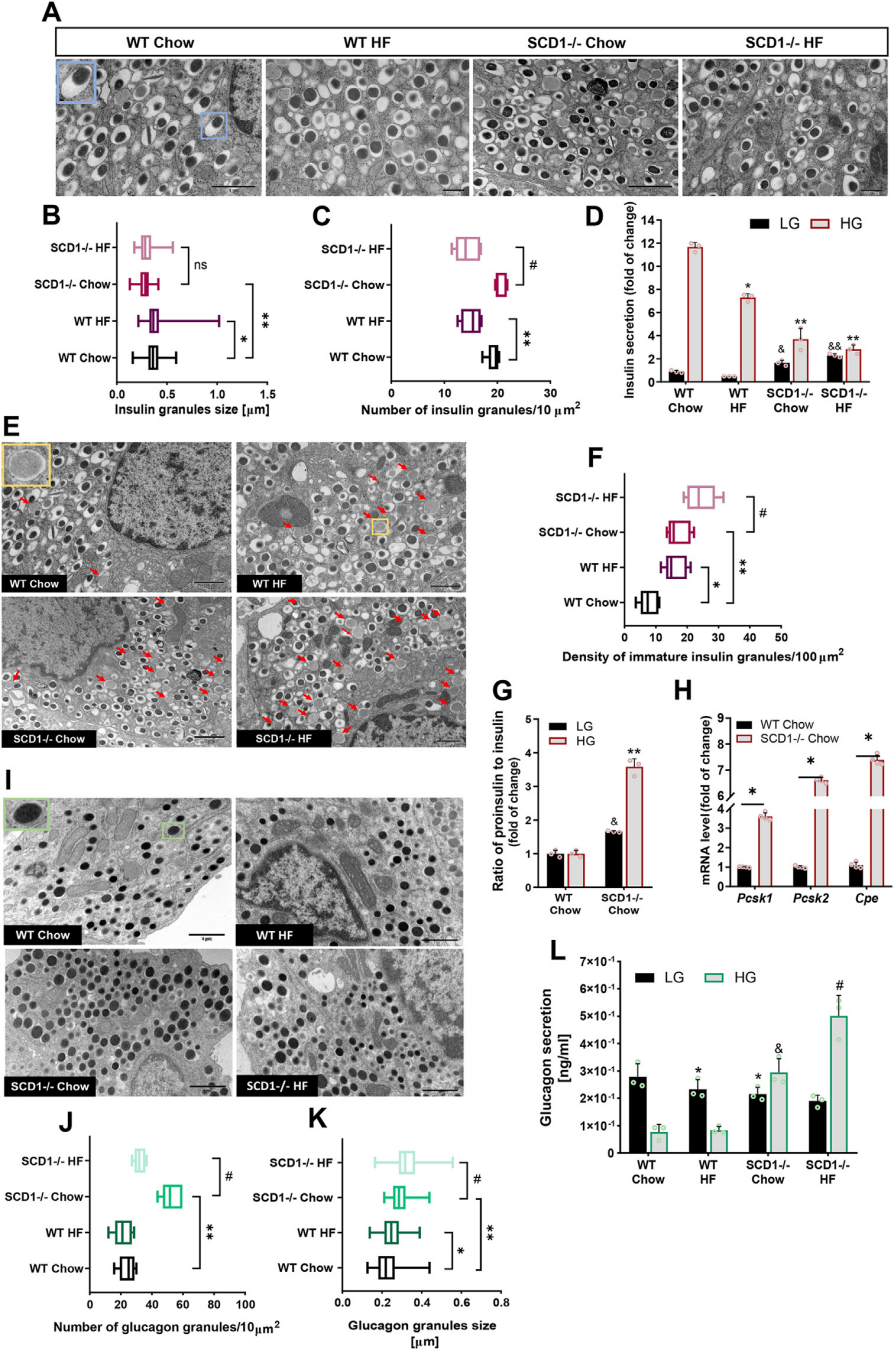
**SCD1−/− knockout in mouse affects the morphology of insulin- and glucagon-containing granules in pancreatic islets, exaggerates basal glucagon release, and disturbs insulin secretion processes. (A, H)** Ultrastructure of insulin **(A)** and glucagon **(I)** secretory granules in individual pancreatic β-cells and α-cells in WT and SCD1−/− mice that were fed a chow or HF diet. Blue-framed and green-framed insets show the morphology of mature insulin granule and glucagon granule, respectively. All images are at the same magnification. **(B, C, J, K)** Size (*n* = 100) and density (*n* = 5) of insulin **(B, C)** and glucagon **(J, K)** secretory granules in the cytoplasm of single β-cells and α-cells. The number of insulin/glucagon granules was calculated per 10 μm^2^ of the cytoplasm. **(E)** Ultrastructure of immature insulin secretory granules in individual β-cells in WT and SCD1−/− mice that were fed a chow or HF diet. Yellow-framed insets show the ultrastructure of immature insulin granule. Red arrows indicate immature insulin granules that were identified in the cell. Density of vesicles that contain proinsulin in the cytoplasm of single β-cells (*n* = 5). The number of immature insulin granules was calculated per 100 μm^2^ of the cytoplasm. *∗p* < 0.05, ∗*∗p* < 0.005, *vs*. WT chow; ^*#*^*p* < 0.05, *vs*. SCD1−/− chow. ns, nonsignificant. **(G)** mRNA levels of *Pcsk1*, *Pcsk2* and *Cpe* genes in pancreatic islets from WT and SCD1−/− mice. *∗p* < 0.05, vs WT Chow. **(D, G, L)** Secretion of insulin **(D)**, proinsulin **(G)**, and glucagon **(L)** from pancreatic islets in WT and SCD1−/− mice that were fed a chow or HF diet. *n* = 3. LG, low glucose concentration (2.75 mM); HG, high glucose concentration (16.5 mM). *∗p* < 0.05, *∗∗p* < 0.005, *vs*. WT chow HG; ^&^*p* < 0.05, *vs*. WT chow LG; *ˆp* < 0.05, *vs*. SCD1−/− chow LG; ^*#*^*p* < 0.05, *vs*. SCD1−/− chow HG. The results are expressed as mean ± SD.

We identified single β-cells that contained mature insulin vesicles, characterized by a central or eccentric high-density core that is surrounded by a wide electron-lucent halo [36] (Figure 6A). The estimated number of insulin secretory granules that formed in WT and SCD1−/− β-cells was similar (Figure 6C), but in SCD1-deficient β-cells, the size of granules was ∼25% smaller than in WT (Figure 6B). Administration of the HF diet reduced the number of insulin-containing granules in SCD1−/− mice by ∼30% compared with their SCD1−/− counterparts that were fed the chow diet (Figure 6B). Consistent with changes in insulin granule morphology, insulin secretion was disrupted in SCD1−/− islets. Pancreatic islets in SCD^−/−^ chow-fed mice that were not stimulated with glucose secreted ∼80% more insulin compared with pancreatic islets in WT chow-fed mice (Figure 6D). The insulin secretory capacity of islets in SCD1−/− chow-fed mice in response to elevated glucose concentration decreased by nearly 70% compared with islets in WT chow-fed mice (Figure 6D). Additionally, pancreatic islets in WT HF diet-fed mice released ∼38% less insulin, and pancreatic islets in SCD1−/− HF diet-fed mice released ∼24% less insulin compared with WT chow-fed mice and SCD1−/− chow-fed mice, respectively (Figure 6D). The number of proinsulin-containing granules was more than two-fold higher in the cytoplasmic area in SCD1−/− β-cells compared with WT β-cells (Figure 6E, F). The frequency of immature insulin vesicles in the cytoplasm of pancreatic islets also increased by ∼40% in β-cells in SCD1−/− HF diet-fed mice compared with SCD1−/− chow-fed mice (Figure 6E, F). Interestingly, SCD1−/− chow islets showed also markedly increased expression of *Pcsk1*, *Pcsk2* and *Cpe* genes (Figure 6H), encoding major prohormone convertases that process proinsulin cleavage [37]. Impairments in β-cell function in pancreatic islets in SCD1−/− chow-fed mice also manifested as an increase in the secretion of proinsulin compared with insulin (>3-fold increase in the presence of HG and ∼65% increase in the presence of LG) compared with islets in WT chow-fed mice (Figure 6G).

To understand the influence of SCD1 on the α-cell population, we also assessed secretory granules in single α-cells in pancreatic islets that were isolated from WT and SCD1−/− mice using TEM. The morphological properties of glucagon-containing granules, visualized by TEM, included a large electron-dense core and a narrow, fitted lucent halo (Figure 6I). The ultrastructural study revealed that the average size of glucagon-secretory granules was 25% higher in SCD1−/− α-cells compared with WT (Figure 6K). Furthermore, SCD1 knockout increased nearly three-fold the compaction of glucagon-containing vesicles in the cytoplasm area of α-cells compared with WT (Figure 6J). Secretory granules in α-cells in WT HF diet-fed mice were ∼8% smaller compared with their chow-fed littermates, and the number of granules that formed in the cytoplasm decreased by ∼12% (Figure 6J, K). SCD1−/− HF diet-fed animals exhibited a ∼12% increase in the mean granule size and ∼40% decrease in the number of glucagon-containing vesicles in the cytoplasm of α-cells compared with SCD1−/− chow-fed animals (Figure 6J, K). The deletion of *Scd1* significantly upregulated basal glucagon secretion (at HG concentration) [38] from α-cells in pancreatic islets. Glucagon release from islets in SCD1−/− chow-fed mice in the presence of HG increased more than three-fold compared with their WT counterparts that were fed the same diet (Figure 6L). Glucagon secretion from islets in SCD1−/− HF diet-fed mice increased by ∼70% in the presence of HG compared with SCD1−/− chow-fed mice (Figure 6L). Interestingly, glucagon release in the presence of HG was even higher than in the presence of LG (by ∼30% from islets in SCD1−/− chow-fed mice and ∼2-fold from islets in SCD1−/− HF diet-fed mice; Figure 6L). Glucagon secretion from islets in WT HF diet-fed mice and SCD1−/− chow-fed mice decreased by ∼15% and ∼20%, respectively, in the presence of LG compared with islets in WT chow-fed mice (Figure 6L).

## 3 Materials and methods

### 3.1 Mouse models

Experimental procedures in this study were conducted using the following animal models: 10-week-old male WT and *Scd1* global knockout (SCD1−/−) mice on an identical C57/BL6 background, pure-bred WT and SCD1−/− homozygous newborn (P0) mice, and WT and SCD1−/− embryos derived on E15.5 or E18.5 of embryonic development. The generation of SCD1−/− knockout mice was previously described [70]. The mice were continuously fed a standard laboratory chow diet (Ssniff, Soest, Germany) with access to water *ad libitum* and maintained under 14-h light conditions. The animals were sacrificed by cervical dislocation.

To generate mouse embryos, males were introduced to females, and then mated females were observed for the presence of vaginal plugs. The day of vaginal plug appearance was considered half of the first pregnancy day. In some cases, mice were reproduced by *in vitro* fertilization and a subsequent embryo transfer. To this end, mouse females were superovulated with an intraperitoneal injection of 7.5 IU of pregnant mare serum gonadotrophin (PMSG, Intervet) followed 48 h later by 7.5 IU of human chorionic gonadotrophin (hCG, Intervet). Epididymal sperm was isolated from male mice and capacitated in fertilization medium with 5 mg/ml BSA [71] for 1.5–2 h in 37.5 °C and 5% CO_2_ in the air. Ovulated oocytes were recovered from oviducts 15 h after hCG and co-incubated with capacitated sperm for 4 h as described in [72]. Fertilized oocytes were transferred to KSOM medium (Speciality Media, Merck Millipore) and cultured for 18 h. Those that reached 2-cell stage were then transferred to oviducts of pseudopregnant WT mouse females (F1 (C57Bl/Tar × CBA/Tar)) on the 1st day of pseudopregnancy.

### 3.2 Cell lines

INS-1E cells (rat insulin-secreting β-cell line) and αTC1-6 cells (mouse glucagon-secreting α-cell line), well-established and widely used pancreatic β- and α-cell surrogates [73,74], were used in this study. Transcriptome of INS-1E and αTC1-6 cells overlap significantly with the transcriptomes of primary mouse/human islet cells. Moreover, INS-1E and αTC1-6 exhibit functional hallmarks of its primary islet cell counterpart and are well characterized in terms of expression of pancreatic β- and α-cell specific TFs, which makes them a suitable model to guide studies on pancreatic islet cell identity [[74], [75], [76]].

INS-1E cells were obtained from Dr. Pierre Maechler (University of Geneva, Geneva, Switzerland). The cells were cultured in complete RPMI medium that was supplemented with 5% heat-inactivated fetal bovine serum (FBS), 1 mM sodium pyruvate, 10 mM HEPES, 2 mM glutamine, 50 μM 2-mercaptoethanol, 100 IU/ml penicillin, and 100 μg/ml streptomycin.

αTC1-6 cells were purchased from ATCC (CRL-2934). αTC1-6 cells were cultured in LG Dulbecco’s modified Eagle’s medium (Gibco) that was supplemented with 10% heat-inactivated FBS, 15 mM HEPES, 0.1 mM nonessential amino acids, 2 mg/ml glucose, 0.02% bovine serum albumin (BSA), 100 IU/ml penicillin, and 100 μg/ml streptomycin. Growth media were changed every 2–3 days. Both INS-1E and αTC1-6 cells were maintained in a 5% CO_2_ atmosphere at 37 °C.

### 3.3 SCD1 inhibition and overexpression and chronic treatments

To inhibit the enzymatic activity of SCD1, the INS-1E and αTC1-6 cells were incubated for 20 h with 2 μM of the SCD1 inhibitor A939572 (Biofine International, Blain, WA, USA). Desaturation indices of palmitic and stearic acids were measured and used as indicators of SCD1 activity (Figure S2) as described previously [23]. Additionally, to induce lipotoxicity, cells were treated with 0.4 mM palmitic acid conjugated with BSA for 16 h before sample collection. Dimethyl sulfoxide (DMSO) and 7.5% BSA were used as vehicle controls for the SCD1 inhibitor and palmitate, respectively.

To transiently overexpress SCD1 in INS-1E and αTC1-6 cells, the cells were transfected with the pCMV6-Entry vector that encoded C-terminally Myc-tagged human SCD1 protein (0.4 μg/cm^2^) using Lipofectamine 2000 reagent (0.8 μl/cm^2^, Thermo Fisher Scientific). Control cells were transfected with pCMV6-Entry empty plasmid. Cells were harvested 24 h after transfection. For each experiment, the intracellular expression of recombinant Myc-tagged SCD1 protein was confirmed by Western blot.

### 3.4 Pancreatic explant/islet collection

The uterus with deciduomas was removed from pregnant females on E15.5 and E18.5. Next, the uterus was incised, and decidual capsules with embryos were exposed. Embryos were released from the amniotic sac by preparation tweezers, washed with phosphate-buffered saline (PBS) and fixed in 4% paraformaldehyde (PFA) at 4 °C overnight. Pancreatic explants from postimplanted embryos and newborn mice were collected and stored at −80 °C for gene expression analyses. At 10 weeks of age, whole pancreatic tissue was excised, weighed, and fixed in 4% PFA at 4 °C overnight, or pancreatic islets were isolated.

Mouse pancreatic islets were isolated from 10-week-old mice by perfusion of the pancreata with 5 ml of ice-cold collagenase (Sigma, St. Louis, MO, USA) at a concentration of 0.23 mg/ml solution in Hank’s Balanced Salt Solution (HBSS) through the clamped pancreatic duct. The pancreata was removed and dissociated in 3 ml of collagenase solution at 37 °C in a water bath for 10–15 min to digest exocrine tissue. After repeated washes using HBSS that contained 10% FBS, islets were collected in a 70 μm cell strainer and separated onto a gradient using a combination of HBSS and Histopaque-1077 (Sigma–Aldrich). Histopaque was then removed from the islet suspension by a series of rinses in HBSS that was supplemented with 10% FBS. To avoid exocrine contamination, islets were hand-picked using a stereomicroscope. Isolated islets were cultured *ex vivo* in non-adherent Petri dishes in RPMI medium that contained 5% FBS, 2 mM glutamine, 100 mg/ml streptomycin, and 100 IU/ml penicillin at 37 °C under a 95% air and 5% CO_2_ atmosphere, or they were stored at −80 °C for further analyses.

### 3.5 Dietary intervention and glucose tolerance test

The mice were fed a HF diet (60% calories from fat; Harlan-Tekland) for 6 weeks. The animals were monitored for weight gain every week. After 6 weeks of the diet, the glucose tolerance test was performed. Male mice (*n* = 3 mice/group) were fasted overnight (∼15 h) with water available *ad libitum*. Blood glucose levels were measured from tail blood using glucose strips with an Optium Xido glucose meter (Abbott, Warsaw, Poland) before the glucose injection (baseline, 0 min) and 15, 30, 60, 90, and 120 min after an intraperitoneal injection of glucose (1.5 mg glucose/g body weight).

### 3.6 Immunofluorescence staining

Paraformaldehyde-fixed embryos and pancreata were embedded in paraffin and sectioned at 4.5 μm. All specimens that were used for immunofluorescence underwent dewaxing with xylene and rehydration in a descending series of ethanol (100%, 95%, 70%, and 50%), followed by incubation for 5 min at room temperature. Antigen retrieval was performed using 0.1 M sodium citrate buffer (pH 6) at 100 °C in a microwave for 15 min. Slides were then cooled to room temperature for 90 min, rinsed twice with PBS, and blocked with buffer that consisted of 10% BSA in PBS for 1.5 h. The samples were then co-incubated with primary antibodies for insulin (Cell Signaling, Hartsfordshire, UK; catalog no. 3014) and glucagon (Abcam, Cambridge, UK; catalog no. ab10988) diluted 1:400 in 1% BSA solution in PBS at 4 °C overnight. The next day, slides were washed four times with PBS and incubated with appropriate secondary antibodies conjugated to Alexa Fluor-568 and Alexa Fluor-488 (Invitrogen, Carlsbad, CA, USA) for 1 h in the dark at room temperature. The slides were then co-stained with DAPI to identify nuclei and closed with coverslips in Dako fluorescence mounting medium (Agilent Technologies, CA, USA). Single pancreatic islets were visualized using an Olympus BX41 microscope (Olympus America, Breiningsville, PA, USA).

### 3.7 Morphometric analyses

For the quantitative morphometric analysis of mice pancreatic islets during fetal, neonatal, and adult life, at least six evenly spaced (150 μm apart) immune-stained sections for insulin and glucagon throughout the entire embryo/pancreas were picked. Digital images of whole pancreas sections were imaged using an Olympus VS110 virtual slide scanning system (Olympus America). The following parameters were measured and calculated using Image J software with appropriate plug-ins: pancreatic islet size, number, and density (per mm^2^), insulin- and glucagon-positive cell areas, and total pancreas area. Pancreatic α/β-cell mass was calculated by multiplying the ratio of the total glucagon/insulin positive area to total pancreatic area by pancreas weight.

### 3.8 Transmission electron microscopy

Pancreatic islets were fixed immediately after isolation in a solution of 2.5% glutaraldehyde and 2% PFA for 1 h at 4 °C. Isolated islets were then rinsed three times in 0.1% cacodylate buffer (Sigma–Aldrich) for 10 min and postfixed in 2% osmium tetroxide (PolyScience, Niles, IL, USA) for 1 h at room temperature. The samples were then dehydrated by incubation in 50% ethanol (10 min), 70% ethanol that contained 1% uranyl acetate (Ted Pella, Redding, CA, USA) to enhance contrast (40 min), 90% ethanol (10 min), 96% ethanol (10 min), and 100% ethanol (15 min). After dehydration, islets were incubated in a mixture of propylene oxide, embedded in Epon resin (Sigma–Aldrich), and placed in gelatin capsules. Embedded samples underwent polymerization at 60 °C for 48 h. Ultrathin sections were cut with a diamond knife on a Leica Ultracut R ultramicrotome (Leica Microsystems, Vienna, Austria) and collected on TEM copper grids (Ted Pella, Redding, CA, USA). Electron micrographs were acquired with a Morada camera on a JEM 1400 transmission electron microscope at 80 kV (JEOL, Tokyo, Japan).

### 3.9 Analysis of insulin, proinsulin, and glucagon secretion

Before the experiments, islets were cultured *ex vivo* overnight. The next day, islets were hand-picked and preincubated in Krebs Ringer bicarbonate buffer (135 mM NaCl, 3.6 mM KCl, 5 mM NaHCO_3_, 0.5 mM MgCl_2_, 10 mM HEPES, 0.1% BSA, and 0.5 mM NaH_2_PO_4_) without glucose for 1 h. Subsequently, equal populations of islets were transferred to fresh Krebs Ringer buffer that contained LG (2.75 mM) or HG (16.5 mM) for 30 min. Incubation media were then collected to determine insulin concentrations using a Rat/Mouse Insulin Enzyme-Linked Immunosorbent Assay (ELISA) Kit (Merck Millipore, Billerica, MA, USA), proinsulin concentrations using a Rat/Mouse Proinsulin ELISA Kit (Mercodia, Uppsala, Sweden), and glucagon concentrations using a Glucagon ELISA Kit, Chemiluminescent (Merck Millipore, Billerica, MA, USA), according to the manufacturers’ instructions.

### 3.10 DNA methylation analysis by bisulfite sequencing

DNA was extracted and purified using the DNeasy Blood and Tissue Kit (Qiagen, Germantown, MD, USA) and subjected to bisulfite conversion using the EpiTect Fast Bisulfite Conversion Kit (Qiagen, Germantown, MD, USA) according to manufacturer’s protocols. Promoter regions (from 0 to −1000 bp upstream of the transcription start site) of the genes were identified after *in silico* analyses using the University of California, Santa Cruz, database (https://genome.ucsc.edu). CpG island prediction and positioning (island size >200 bp, observed/expected ratio >0.60, percent GC dinucleotides >50.0) and the designation of methylation-specific primers were performed using MethPrimer (https://www.urogene.org/methprimer) and Bisulfite Primer Seeker (https://www.zymoresearch.com/pages/bisulfite-primer-seeker). Selected promoter regions of the *Pdx1* and *MafA* genes were amplified on bisulfite-treated DNA templates with the primers that are listed in Table S2. Polymerase chain reactions (PCRs) were performed with FastStart Taq DNA Polymerase (Roche) in a heated-lid thermocycler under the following conditions: (95 °C, 8 min), (95 °C, 30 s) (57 °C, 40 s) (72 °C, 1.5 min) × 10, (95 °C, 20 s) (57 °C, 30 s) (72 °C, 1 min) × 25, and (72 °C, 10 min). The obtained PCR products were separated and assessed on 1% agarose gel. The specific band products with appropriate sizes were extracted and purified from the gel with Gel-Out Concentrator (A&A Biotechnology, Gdynia, Poland) and then ligated into the pGEM-T Easy pre-linearized Vector System (Promega) that contains 3′-T overhangs. After transformation, recombinant clones were selected by blue/white screening on indicator plates. The presence of inserts in selected clones was confirmed by EcoRI restriction enzyme digestion. The chosen constructs were sequenced using the Sanger method. Promoter sequences for gene promoters were analyzed using BiQ Analyzer 3.0.

### 3.11 RNA extraction and qRT-PCR analysis

For quantitative real-time PCR (qRT-PCR) analysis, total RNA was extracted from the experimental samples using Total RNA Mini Plus Concentrator (A&A Biotechnology, Gdynia, Poland) according to the manufacturer’s protocol. One microgram of DNase-treated RNA was reverse-transcribed using the RevertAid First Strand cDNA Synthesis Kit (Thermo Scientific, Pittsburgh, PA, USA). Triplicate samples for quantitative PCR were run in a CFX Connect Real-Time PCR Detection System (Bio-Rad, Hercules, CA, USA). SsoAdvanced Universal SYBR Supermix (Bio-Rad, Hercules, CA, USA) was used for detection. mRNA expression in each sample was determined after normalization to *Gapdh*, 18*S* ribosomal RNA, or *Hprt*. Gene expression values are expressed as fold changes relative to the control using the ΔΔCt method. Sequences of primers that were used for qRT-PCR are listed in Table S1.

### 3.12 Western blot analysis

Experimental samples were lysed, and protein concentrations in islets and cell lysates were quantified as described previously [24]. Pancreatic islets (15 μg of protein) or INS-1E and αTC1-6 cells (30 μg of protein) were separated by electrophoresis on 10% sodium dodecyl sulfate-polyacrylamide gels, transferred to a polyvinylidene fluoride (PVDF) membrane (Millipore, Billerica, MA, USA), and blotted using appropriate antibodies. The following primary antibodies were used for Western blot: PDX1 (catalog no. 5679, Cell Signaling, Hartsfordshire, UK), FOXO1 (catalog no. 2880, Cell Signaling), anti-Myc Taq (catalog no. 2272, Cell Signaling), NKX2.2 (catalog no. sc-398951, Santa Cruz Biotechnology, Santa Cruz, CA, USA), ISL1 (catalog no. sc-390793, Santa Cruz Biotechnology), PAX6 (catalog no. sc-81649, Santa Cruz Biotechnology), ARX (catalog no. sc-293449, Santa Cruz Biotechnology), glucagon (catalog no. sc-514592, Santa Cruz Biotechnology), and β-actin (catalog no. 3854, Sigma–Aldrich, St. Louis, MO, USA). Chemiluminescent detection was performed using secondary peroxidase-conjugated goat anti-rabbit IgG (catalog no. 67437, MP Biomedicals, Irvine, CA, USA) and goat anti-mouse IgG (catalog no. 115-035-146, Jackson Immuno Research Laboratories, West Grove, PA, USA). Proteins were visualized using SuperSignal West Pico PLUS Chemiluminescent Substrate (Thermo Scientific). Protein levels were quantified by densitometry with reference to β-actin.

### 3.13 Statistical analysis

Statistical significance was assessed using GraphPad Prism 8.3.0 software. Multiple comparisons were performed using one-way analysis of variance (ANOVA) followed by Tukey’s *post hoc* test. Unpaired *t*-tests were used when two groups were compared. Values of *p* < 0.05 were considered statistically significant. The experiments were performed in triplicate unless stated otherwise. The data are expressed as mean ± SD.

## 4 Discussion

The loss of β-cell identity, β-cell dedifferentiation, and the acquisition of multi-hormonal cells are increasingly recognized in pancreatic islets in patients with T2D [7,16]. A growing body of evidence suggests that metabolic stress, particularly hyperglycemia, may compromise the identity of β-cells [5,7]. However, the pathway by which these reprogramming events occur and their participation in disease initiation and progression are still unclear. In the present study, we uncovered the essential role of SCD1 in the repression of α-cell programs during embryonic development of the pancreas and in maintenance of β-cell identity and proper functioning of pancreatic islets in adulthood.

It has been shown that SCD1 is abundantly expressed in human fetal pancreatic α- and β-cells, and its expression further increases in mature α- and β-cells [39]. The lack of SCD1 is related to the chronic shortage of MUFAs and oversupply of SFAs, which is extremely harmful to pancreatic β-cells [40,41]. Fatty acids play diverse roles in developing mammalian oocytes and early embryos [42]. Several lines of evidence have shown that fatty acid exposure during pregnancy affects the structure and function of newly formed pancreatic islets [42,43]. Palmitate (16:0) at high concentrations is associated with long-term metabolic perturbation, leaving the newborn predisposed to diabetes and obesity [44]. Pluripotent cells depend on oleate metabolism for their viability and suppression of SCD1 can lead to the selective elimination of pluripotent cells *in vitro* [45]. Furthermore, immediate requirement for SCD1 activity in the endoderm commitment of pluripotent stem cells may be of importance in disorders of endoderm-derived organs such as pancreas [46]. Here, we demonstrated that the loss of SCD1 on E15.5 was manifested by the higher expression of an α-cell key gene, *Arx*, and lower expression of the β-cell-specific marker *Pax4* in the mouse pancreas, which were followed by the expanded formation of α-cells and delayed formation of β-cells in pancreatic islets. Reciprocal interactions between ARX and PAX4 determine the specification of common progenitors into α-cells or β-cells during pancreas morphogenesis [10,14]. SCD1 deficiency disrupted the dynamics of pancreatic α-cell and β-cell neogenesis on E15.5. However, despite the persistent overexpression of *Arx* from E15.5 to P0 in the pancreas in SCD1−/− mice, expression of the β-cell marker *Pdx1* and endocrine cell marker *Ngn3* and the ratio of α-cell area to β-cell area remained unaltered on E18.5 and in the newborn SCD1−/− pancreas. We also found that newborn WT and SCD1−/− islets shared common architectural features. Deleterious effects of SCD1 deficiency on pancreatic β-cells were previously reported by us and others [22,23,47,48], raising the possibility that pancreatic islets in SCD1−/− mice become dysfunctional in adulthood. Therefore, SCD1 could be involved in the loss of mature β-cell identity.

Previous studies have also shown that metabolic stress, such as lipotoxicity, predisposes to mature β-cell dedifferentiation and reprogramming [5]. SCD1 serves as a major enzyme that protects against 16:0 toxicity in β-cells [22]. The downregulation of SCD1 aggravates and overexpression of SCD1 reduces apoptosis and endoplasmic reticulum stress that is induced by 16:0 in multiple β-cell lines and human pancreatic islets [22,34,49,50]. Our further research showed that metabolic defects that were caused by SCD1 deficiency resulted in the downregulation of β-cell-enriched genes, including *Pdx1*, *MafA*, *Pax6*, *Nkx2.2*, *Isl1*, and *Nkx6.1*, in mature mouse pancreatic islets and rat INS-1E cells. Moreover, SCD1 ablation promoted the decay of *Foxo1* and *Ngn3* mRNA-encoded proteins, which are relevant to β-cell viability, proliferation, and recovery after injury [51,52]. A decrease in the expression of PDX1, MAFA, and ISL1 contributes to the lower expression of insulin through a reduction of the binding of these TFs to the proximal promoter and enhancer sequence of the *Ins* gene [53,54]. Thus, consistent with previous studies, impairments in the expression of Pdx1, MafA, and Isl1 may underlie the disruption of *Ins* transcription and a decrease in glucose-stimulated insulin secretion (GSIS) in β-cells upon the absence of SCD1 [22,23]. The present results also indicate that SFA desaturation is part of the mechanism by which SCD1 protects β-cells from lipotoxicity. We demonstrated that stimulation with 16:0 exacerbated the reduction of expression of PDX1, MAFA, and ISL1 in SCD1-deficient rat β-cells, thereby accentuating metabolic stress that is caused by SCD1 inhibition. MafA, Pax6, and Nkx2.2 also act as repressors of several β-cell-disallowed genes [17,18,55]. The abundance of these repressors significantly decreased in SCD1−/− mouse pancreatic islets. Upregulation of the β-cell progenitor marker *Sox9* in SCD1−/− islets, likely as a result of the downregulation of β-cell-specific TFs, indicated a failure to attain a fully differentiated state of β-cells in mouse pancreatic islets. A similar conclusion was reached in an independent study by Oshima et al. Gene expression analyses showed that the EndoC-βH1 human pancreatic β-cell line with *Scd1* knockout exhibited the lower expression of a number of genes that encode TFs that determine the maintenance of mature β-cell identity (i.e. *INS, PDX1, MAFA, FOXO1*, *SLC30A8*). Moreover, induction of the expression of β-cell differentiation markers (i.e., *SOX9*, *MYC*, and *HES1*) was observed in SCD1−/− EndoC-βH1 cells that were incubated with 16:0 [22]. Interestingly, the TF SOX9 was also upregulated in pancreatic islets from T2D donors [56]. In contrast to SCD1 inhibition, the overexpression of SCD1 in INS-1E cells led to an increase in the expression of such TFs as Pdx1, Pax6, Foxo1, and Nkx2.2, supporting the hypothesis that SCD1 acts as metabolic checkpoint in the maintenance of mature β-cell identity.

DNA methylation is generally essential for β-cell maturation and contributes to the repression of immature β-cell genes [57]. Our recent research showed that SCD1 activity preserves DNMT1-mediated DNA methylation patterns in pancreatic β-cells. We found that SCD1 inhibition/deficiency caused DNA hypomethylation and changed the methyl group distribution within chromosomes in β-cells [24]. Additionally, in the present study, we discovered the involvement of SCD1 in the regulation of the promoter methylation status of crucial β-cell genes, such as *Pxd1* and *MafA*. The hypermethylation of *Pdx1* and *MafA* promoters was related to a decrease in the expression of these TFs under conditions of SCD1 deficiency and 16:0 treatment in INS-1E cells. Therefore, SCD1 may participate in the control of adult β-cell identity via epigenetic mechanisms.

In the present study, we also considered the contribution of SCD1 to regulation of the expression of TFs that are responsible for the maintenance of mature α-cell identity. To our knowledge, there are no previous data that describe the role of SCD1 in controlling α-cell fate. Contrary to INS-1E cells, SCD1 inhibition, SCD1 overexpression, and 16:0 did not influence the expression levels of α-cell markers (Foxo1, Pax6, Nkx2.2, and Arx) in αTC1-6 cells. Pancreatic islets in SCD1−/− mice exhibited an increase in the expression of α-cell-specific genes (i.e., *Arx* and *MafB*), likely because of an increase in α-cell number within these islets (discussed in the sections below). Our analyses did not reveal any symptoms that indicated a potential propensity for α-cell dedifferentiation or transdifferentiation that was caused by SCD1 inhibition. The influence of SCD1 substrates and products on the accumulation of lipid metabolites and lipid signaling in α-cells is still not clearly defined [58,59]. Nonetheless, the lower plasticity of α-cells under conditions of SCD1 deficiency could potentially result from the greater resistance of α-cells to palmitate-induced lipotoxicity [60].

Alterations of the expression of α-cell- and β-cell-specific TFs that were present in SCD1-deficient mouse pancreatic islets and β-cells were reflected by pancreatic islet cytoarchitecture and function. Several abnormalities were identified in pancreatic islets in SCD1−/− mice. These included decreases in β-cell mass and the size of insulin secretory granules, followed by lower GSIS. Islets in SCD1−/− mice were also characterized by an increase in the number of proinsulin-containing granules, higher expression of genes encoding prohormone convertases responsible for C-peptide release, and an increase in the secretion of insulin under low-glucose conditions compared with WT. This indicates that SCD1 deficiency may affect glucose sensing and impairs the machinery of insulin synthesis and secretion in pancreatic β-cells. A decrease in granularity and an increase in the secretion of insulin upon low-glucose stimulation are typical for immature β-cells and could also be considered hallmarks of β-cell dedifferentiation [57,61]. Nevertheless, our study did not unravel the presence of single, hybrid β-cells that acquired multihormonal features and symptoms of β-cell to α-cell transdifferentiation in pancreatic islets in SCD1−/− mice.

In contrast, SCD1 knockout in the mouse led to expansion of the α-cell population within islets, an increase in the biosynthesis of glucagon-containing granules in α-cells, and greater glucagon release from α-cells under conditions of HG concentrations (i.e., when glucagon secretion is inhibited [38]). Moreover, in pancreatic islets in SCD1−/− mice, α-cells were also present in the core of islets, which differs from the classic peripheral localization of these cells in mouse islets of Langerhans [62]. The β-cell function is regulated by paracrine crosstalk with other endocrine cell types in the islet, notably the α-cells and somatostatin-producing δ-cells [63]. While the α-cell releases factors that are stimulatory for β-cell and increase GSIS, the products of the β-cell inhibit α-cell function [64]. The perturbation of intra-islet stimulation of β-cells by glucagon from α-cells could contribute to marked elevation of basal insulin release from SCD1−/− knock-out islets.

Disturbances in the regulation of pancreatic hormone release upon SCD1 knockout resulted in an improper response of SCD1-deficient β- and α-cells to metabolic stress. Administration of the HF diet in SCD1−/− mice was related to similar but much more severe defects in pancreatic islet architecture and function compared with WT. The HF diet-induced obesity further aggravated β-cell dysfunction in SCD1-deficient islets; however HF diet did not induce weight gain or insulin resistance in SCD1−/− mice. The simultaneous occurrence of β-cell dysfunction and beneficial systemic effects of global *Scd1* gene knockout in mice has been reported previously [19,20]. Mice with the targeted deletion of *Scd1* are characterized by a lean hypermetabolic phenotype that includes a lower rate of lipogenesis, a decrease in the accumulation of triglyceride and cholesterol esters, an increase in β-oxidation in liver, muscle, and adipose tissue [19], an increase in insulin signaling in muscle and adipose tissue [65,66], and significant changes in nonshivering thermogenesis [67]. Glucagon, similar to insulin, has been shown to control FA metabolism in peripheral tissues [68]. Glucagon stimulates lipolysis in adipose tissue and promotes FA β-oxidation and inhibits lipogenesis in the liver [68]. The strong basal overproduction of glucagon by pancreatic islets in SCD1−/− mice may partially explain the overall systemic effects and lean phenotype that is observed in SCD1−/− mice. SCD1 might be a promising therapeutic target for the chronic treatment of diabetes and dyslipidemia [69]. The present data emphasize the necessity to design targeted SCD1 inhibitors that avoid adverse effects that can be caused by the systemic inhibition of SCD1, such as pancreatic islet dysfunction.

In conclusion, the present study identified SCD1 as an important regulator of β-cell identity and function. The present results indicate that SCD1 controls pancreatic islet organogenesis, determines the balance between insulin and glucagon secretion, regulates the expression of key β-cell genes via epigenetic mechanisms, and protects β-cells from the lipid-derived loss of identity. Our study sheds more light on the molecular nature of processes of β-cell failure and selective α-cell expansion that contribute to pancreatic islet dysfunction in T2D. These findings provide additional mechanistic insights into the role of SCD1 in T2D pathogenesis and may contribute to the discovery of effective therapeutic approaches for T2D.

## Funding

This work was supported by grants from the 10.13039/501100004442National Science Centre, Poland (no. UMO-2017/27/N/NZ3/01987 to A.M.D., UMO-2013/10/E/NZ3/00670 to A. Do., and UMO-2015/19/D/NZ4/03705 to J.J.) and National Centre for Research and Development (no. STRATEGMED 3/305813/2/NCBR/2017 to A. Do.).

## Ethics approval

All animal protocols in this study were approved by the regional authority (First Local Ethical Committee for Animal Experiments in Warsaw, permit no. 37/2016 and 205/2016).

## Author Contributions

A.Do. and A.M.D. conceptualized and designed the research. A.M.D., J.J., A.Dz, D.W., H.N., A.A.Sz. and E.K. were involved in experimentation. A.A. and M.Sz. performed in vitro fertilization and embryo transfers. All authors contributed to the data analysis and interpretation. A.M.D. and A.Do. wrote and proofread the manuscript. All authors approved the final version of the manuscript.

## Figures and Tables

**Supplementary Figure 1:**
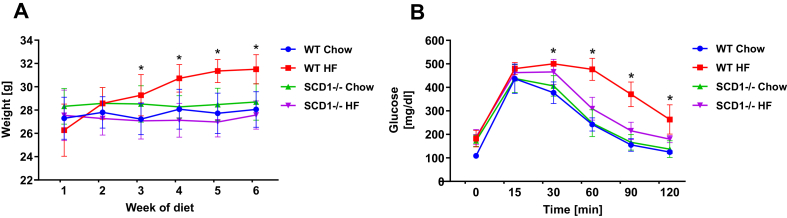


**Supplementary Figure 1:**
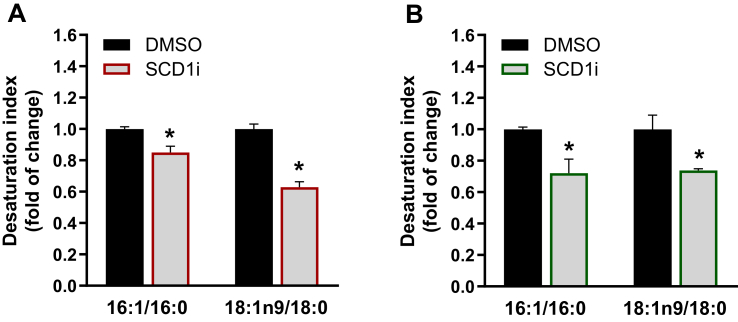


## Data Availability

No data was used for the research described in the article.

## Footnotes

Supplementary data to this article can be found online at https://doi.org/10.1016/j.molmet.2022.101659.
