# Supplementary material for: Inhibition of stearoyl-CoA desaturase 1 in the mouse impairs pancreatic islet morphogenesis and promotes loss of β-cell identity and α-cell expansion in the mature pancreas
Source: Mol Metab. 2022 Dec 15;67:101659. doi: 10.1016/j.molmet.2022.101659 (PMC9801219; doi:10.1016/j.molmet.2022.101659)
Supplement: Multimedia component 1 [file mmc1.docx]

**Inhibition of stearoyl-CoA desaturase 1 in the mouse impairs pancreatic islet morphogenesis and promotes loss of β-cell identity and α-cell expansion in the mature pancreas**

*Dobosz A. M. et al.*

**Supplementary Material**

**Figure S1**

**Figure S1. Response of WT and SCD1-/- mice on a HF diet. (A)** Body weight of WT and SCD1-/- mice that were fed a standard laboratory diet (Chow) or diet enriched in fat (HF) for 6 weeks. **(B)** Results of intraperitoneal glucose tolerance test in WT and SCD1-/- mice that were fed a chow or HF diet. The data are representative of *n* = 6 mice/group. The results are expressed as mean ± SD. **p* < 0.05, *vs*. WT chow.

**Figure S2**


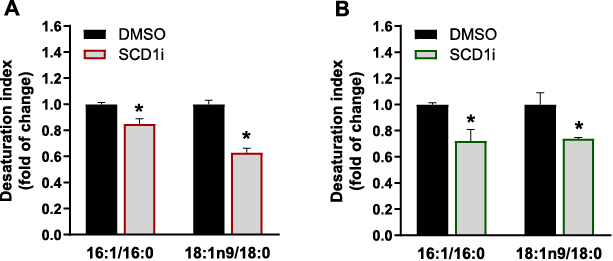


**Figure S2. Evaluation of SCD1 activity after treating pancreatic αTC1-6 and INS-1E cells with 2 μM of the SCD1 inhibitor A939572 (SCD1i).** **(A, B)** Desaturation ratio of 16:0 and 18:0 FAs in total lipid extracts from INS-1E cells **(A)** and αTC1-6 cells **(B)**. The data are expressed as mean ± SD. The data are representative of *n* = 3 independent samples. **p* < 0.05, *vs*. DMSO.

**Table S1.** Real-time PCR primers list.

| Gene | Forward primer | Reverse primer |
| --- | --- | --- |
| *18S rRNA* | 5’TCACCATCATGCAGAACCCA3’ | 5’CCTGGCTGTACTTCCCATCCT3’ |
| *Arx* | 5’TCCGGATACCCCACTTAGCTT3’ | 5’GACGCCCCTTTCCTTTAAGTG3’ |
| *Cpe* | 5’CGGTCCTAACAATCACCTGCTG3’ | 5’ACAAGGTCTCCTCCGTGCAGAT3’ |
| *FoxO1* | 5’TTTGTCACGATGGAGGTGGC3’ | 5’GCCAAAGAGAACAGAGGTGGG3’ |
| *Gapdh* | 5’ACCCAGAAGACTGTGGATGG3’ | 5’CACATTGGGGGTAGGAACAC3’ |
| *Gcg* | 5’AAGAGGAACCGGAACAACATTG3’ | 5’GCCCTCCAAGTAAGAACTCACA3’ |
| *Hprt* | 5’AGTCCCAGCGTCGTGATTAG3’ | 5’TTTCCAAATCCTCGGCATAATGA3’ |
| *Ins1* | 5’CCTTTGTGGTCCTCACCTGG3’ | 5’CGACGGGACTTGGGTGTGTA3’ |
| *Ins2* | 5’CCAGGCTTTTGTCAAACAGCA3’ | 5’AAGAATCCACGCTCCCCACA3’ |
| *Isl1* | 5’TACGTGCTTTGTTAGGGATGGG3’ | 5’CTGCACTTGGCGCATTTGAT3’ |
| *MafA* | 5’CTTCAGCAAGGAGGAGGTCAT3’ | 5’TTCTCGCTCTCCAGAATGTGC3’ |
| *MafB* | 5’GCAACGGTAGTGTGGAGGAC3’ | 5’TTCAGGCGGATCACCTCGT3’ |
| *Neurod1* | 5’ATGACCAAATCATACAGCGAGAG3’ | 5’TCTGCCTCGTGTTCCTCGT3’ |
| *Ngn3* | 5’CCTCTGGGTCTCATCACTGC3’ | 5’TCGCCTGGAGTAAATTGCGT3’ |
| *Nkx2.2* | 5’GAGTCACCGGACAATGACAAG3’ | 5’TAGGTCTGCGCTTTGGAGAAG3’ |
| *Nkx6.1* | 5’CGCTTGGCCTATTCTCTGGG3’ | 5’CTGCGTGCTTCTTTCTCCA3’ |
| *Pax6* | 5’TAGCCCAGTATAAACGGGAGTG3’ | 5’CCAGGTTGCGAAGAACTCTG3’ |
| *Pcsk1* | 5’GGAGAGAATCCTGTAGGCACCT3’ | 5’GCTCTGGTTGAGAAGATGTCCC3’ |
| *Pcsk2* | 5’ACCTCTTTGGCTACGGAGTCCT3’ | 5’TTGAGGGTCAGTACCAGCTTGC3’ |
| *Pdx1* | 5’TTCCCGAATGGAACCGAGC3’ | 5’GCGTGAGCTTTGGTGGATT3’ |
| *Sox9* | 5’CGGAACAGACTCACATCTCTCC3’ | 5’GCTTGCACGTCGGTTTTGG3’ |

**Table S2.** Bisulfite primers list.

| Gene | Forward primer | Reverse primer |
| --- | --- | --- |
| *Pdx1* | 5’ATTGAAGTAATTAATTT-AAAGGATAGTGTA3’ | 5’AAAAACTACAAACCAAACCTTAAAC3’ |
| *MafA* | 5’TAGGYGTTTAGAGATATTA-GTTTTATGTATTTAATGGG3’ | 5’ATCTTACRCTCCTCCTCCTACTCCTC3’ |
